# A standardized criteria-based progressive shoulder exercise program is effective in managing rotator cuff-related shoulder pain: A prospective cohort study

**DOI:** 10.1371/journal.pone.0328728

**Published:** 2025-07-23

**Authors:** Judy Chepeha, Anelise Silveira, David Sheps, Charlene Luciak-Corea, Fiona Styles-Tripp, Lauren Beaupre

**Affiliations:** 1 University of Alberta, Collaborative Orthopaedic Research, Edmonton, Alberta, Canada; 2 University of Alberta, Faculty of Rehabilitation Medicine, Edmonton, Alberta, Canada; Carol Davila University of Medicine and Pharmacy: Universitatea de Medicina si Farmacie Carol Davila din Bucuresti, ROMANIA

## Abstract

**Background:**

Non-operative management of patients with rotator cuff related shoulder pain (RCRSP) has been shown to be effective with outcomes similar to operative interventions. Exercise therapy and education are recommended as first-line treatments; however, program content, progression and referral criteria are typically inadequately detailed to be easily reproduced by clinicians. This study evaluated the effectiveness of a well-defined, criteria-based progressive exercise physical therapy (PT) program in patients with rotator cuff related shoulder pain (RCRSP) over a 26-week evaluation period.

**Methods and findings:**

A longitudinal, prospective cohort study evaluated 143 patients aged 30–65 years with RCRSP. Participants participated in a 12-week in-person and home-exercise shoulder program and were assessed at baseline, 6-weeks, 12-weeks and 26-weeks. Primary outcome measures were pain at rest, at night and with activity. Secondary measures were active range of motion (ROM), strength, and health related quality of life (HRQL) using the Western Ontario Rotator Cuff (WORC) score.

**Results:**

Significant reductions in pain at rest, at night and with activity (p < 0.001) occurred within six weeks and continued to 26-weeks. Active range of motion (ROM), particularly abduction and external rotation in 90 degrees of abduction, significantly improved between 0- and 6-weeks (p < 0.001) as well as at 12-weeks (p < 0.001) and 26-weeks (p < 0.001). Strength similarly improved, especially between 6- and 12-weeks (p < 0.001). Ongoing improvements were reported at 26-weeks. Finally, the WORC score improved over time, with significant improvements at each measurement point, and clinically important improvements occurring within 12 weeks.

**Conclusion(s):**

Utilizing a criteria-based progressive PT program that provided detailed information, including progression criteria and a proven algorithm for referring non-responsive patients for an orthopedic surgical consult was effective in significantly reducing pain, improving active ROM, strength and HRQL in those living with RCRSP.

## Introduction

Shoulder pain and its resultant dysfunction are common patient complaints with rotator cuff (RC) pathology constituting up to 85% of shoulder complaints treated by health professionals [1–4]. Incidence increases with age from about 30% when one is in their sixties to 65% in their eighties [1–4]. With a rapidly aging population and increased participation of this population in the workforce, the projected incidence and consequence of RC related disorders is likely to increase [1–4].

Rotator cuff-relative shoulder pain (RCRSP) encompasses a broad range of shoulder diagnoses including rotator cuff tendinopathy, subacromial impingement syndrome and/or subacromial pain syndrome [5]. Symptoms are described as pain, primarily in the anterolateral shoulder and challenges with shoulder abduction and rotation mobility and strength. Structures within the subacromial space, proximal humerus, rotator cuff tendons and bursa are all believed to contribute to the overall pain response [5]. The cause of RCRSP is multifactorial including age, loading history, biomechanical factors, psychological factors, lifestyle and general health [5]. Treatment goals for patients with (RCRSP) include pain management, improved shoulder range of motion (ROM), strength, and restored upper extremity function for work, recreation and daily activities [5,6]. Strategies to achieve these goals have been well examined with positive outcomes from both non-operative and operative management [5–15]; however, questions remain regarding which interventions are most appropriate based on patient presentation*.* Studies have shown that non-operative management is effective in the large majority of patients with RCRSP [6–9,11,14,16–18], with exercise therapy and education recommended as first-line treatment interventions [19–22]. Less is known regarding the specific program content and delivery method of physical therapy (PT) programs as well as milestones used for program progression [6,20–23].Without detailed reporting of program content and evaluation, it is difficult for clinicians to replicate and measure/compare the effectiveness of varying rehabilitation programs.

This longitudinal prospective cohort study followed patients presenting with RCRSP to determine the impact of a 12-week, standardized evidence-informed, consensus-derived progressive exercise PT program that included both supervised sessions and a home exercise program over a 26-week evaluation period. Emphasis was placed on delivering a detailed, standardized program that allowed customization of dosage, intensity and progression timelines based on individual patient presentation and response. We previously reported that most study participants improved their Western Ontario Rotator Cuff (WORC) score ≥15% with treatment and <12% required surgery when following this program [16].

In this current assessment of recovery over time, our primary research objective was to determine, for patients with primarily RCRSP, the improvements in pain (at rest, at night, and with activity) measured by a numeric rating scale (primary outcome), active shoulder ROM using a universal goniometer, strength measured using a handheld dynamometer, and health-related quality of life (HRQL) as measured by the WORC score over 26-weeks.

## Materials and methods

### Study design

We recruited participants between 2015 and 2018 in a metropolitan Canadian city, and followed them for 26-weeks after recruitment, using a prospective cohort design. The study received approval from the University of Alberta Health Research Ethics Panel (PRO00052670) with all participants providing signed informed consent. The study sample size calculation was based on the primary study question, which was previously published [16]. For the secondary longitudinal analyses described here, we reported our results relative to established minimally clinically important differences (MCID) in our outcome measures over time or in effect sizes of treatment magnitude at 26-weeks relative to baseline for outcomes where no published MCIDs exist. We followed the STROBE reporting guidelines [24].

### Selection criteria

Patients with RCRSP, no PT in the past 12 weeks, between 30–65 years of age, English-speaking and who were willing and able to provide consent and return for follow-up visits were considered eligible. The diagnosis of RCRSP was derived through typical history and examination procedures conducted by primary care providers (PTs, physicians). Potential participants had complaints of anterolateral shoulder pain worsened by elevation above 90 degrees, especially in abduction, as well as weakness and pain most markedly in external rotation and abduction. We excluded those with shoulder instability, advanced joint arthropathy (proximal humeral migration, acromial erosion and/or joint arthrosis), recent shoulder fracture(s), inflammatory arthropathy, or infection.

### Enrolment

Primary care providers (PTs, physicians) identified potential participants who were contacted by the research associates; in Canada, patients are able to seek PT without requiring a physician referral. After confirming eligibility, the research associate obtained signed informed consent from willing volunteers. We then collected demographics (age, sex), comorbidities, medications, injury characteristics (e.g., onset type; symptom duration) and work characteristics for those who were working (employment status, work type, work-related injury). Diagnostic ultrasound was used to identify the proportion of patients with imaging-confirmed rotator cuff pathology, but all patients with RCRSP who met the selection criteria were included in the study as evaluation of diagnostic imaging results was not performed until the study was completed. This allowed evaluation of whether knowledge of shoulder pathology pre-intervention affected treatment response. Participants completed pain visual analogue scales (VAS) and the WORC score while the research associate assessed shoulder active ROM using a goniometer and shoulder strength using a handheld dynamometer. The research assessor did not provide the PT intervention.

### Intervention

All participants completed a standardized PT regime that included supervised PT and a home program for up to 12-weeks or as needed to relieve participant symptoms or demonstrate lack of progression. Participating PTs attended a training session where they received the non-operative shoulder guidelines and were given access to online resources to ensure understanding of the PT regime and program delivery, including criteria for progression.

The exercise program was based on an evidence-informed and consensus-derived 3-phase Shoulder Guideline, with each phase having three sections: 1) Goals, 2) Specific Treatment Interventions and 3) Criteria for Progression. Key principles of patient education, managing pain, and improving shoulder and trunk mobility and strength, with a focus on functional movement patterns, were incorporated.

The program was similar to previously published guidelines that were also based on limited evidence for effectively treating RCRSP [18] as well as those developed by our multidisciplinary Shoulder and Upper Extremity Research Group of Edmonton (SURGE).

Participants and PTs determined the frequency and number of supervised PT sessions based on participant’s symptoms and ability to attend PT. All participants were provided a home exercise program and were directed to complete the program at least four times per week. Dosage parameters were standardized at three sets of each exercise with repetitions determined individually as the number successfully completed with good technique, without sharp pain, and to fatigue. Patients were taught how to use the modified Borg rate of perceived exertion (RPE) scale that measures exertion on a scale of zero (no exertion or resting) to ten (maximum effort) and advised to aim between four and six out of ten (moderate to somewhat hard, high) [25]. Education regarding pain expectation was also delivered with the advice of working “to but not through” pain and to aim for descriptions of “discomfort” or “feeling it” rather than “pain”.

Typically, participants attended PT every 1–2 weeks initially with session frequency decreasing over time as patients became familiar with the exercise program. PTs assessed participants’ shoulder pain (at rest, at night, with activity), active ROM, strength and exercise program tolerance as per a referral algorithm at 2-, 6- and 12-weeks. This algorithm was concurrently tested with the evaluation of program impact and aimed to identify patients who might benefit from a surgical consultation based on non-response to treatment [16]. (Appendix S1)

### Follow-up assessments

An independent research assessor re-evaluated pain, active shoulder ROM, strength, and compliance with the exercise program at 6-, 12-, and 26-weeks after beginning the PT program; participants also completed the WORC at each visit.

### Outcomes/outcome measures

The primary outcome was **pain at rest, at night and with activity** measured using an 11-point VAS (0–10), a reliable and valid method of measuring patient-reported pain [26] with a MCID reported at 1.4 [27].

The secondary outcomes included were:

**Active shoulder flexion, abduction, external rotation (neutral abduction, 90° elbow flexion), and internal and external rotation (90° abduction, 90° elbow flexion)** that were measured by the research assessor using a universal goniometer, a reliable approach to assess joint ROM [28,29].

**Peak values of isometric shoulder flexion, abduction, external rotation and internal rotation** that were measured with the patient’s arm in neutral (neutral abduction, 90° elbow flexion) using a handheld dynamometer (microFET3, Hoggan Health Industries Inc., West Jordan, UT) a valid and reliable strength measure [30,31].

**WORC Score,** a 5-part (physical symptoms, sports/recreation, work, lifestyle, emotions), 21-item, disease-specific questionnaire [32] that is valid, reliable and responsive in the RC patient population [33]. The WORC was selected as the large proportion of participants had image-confirmed RC pathology, and advanced arthritis and instability-related syndromes were excluded. The WORC measures overall shoulder pain and function with a 12% change reflecting the MCID [33,34].

Compliance with the exercise program was assessed through patients’ self-report on attending PT and home exercise completion as well as having participants demonstrate their exercises for the research assessor, who was not involved in delivering the intervention. This allowed the assessor to determine if participants (1) knew their exercises well enough to demonstrate them and (2) were performing the exercises with correct technique and proper dosage parameters.

Demographic and medical characteristics included age, sex, comorbidities and medication use (i.e., NSAIDS, opioids, previous shoulder injections) while shoulder injury information determined type of onset (atraumatic vs traumatic), duration of symptoms, whether the dominant side was affected and whether it was a work-related injury. We also asked employment status (full-time/part-time vs Retired/Student/Unemployed) and when participants were working, their typical workload (Repetitive above shoulder, Repetitive at shoulder, Repetitive at waist, Desk job, No repetitive activity). If participants had diagnostic imaging performed within the past year, those results were used, and the participant was considered to be aware of their results. Those who had not had diagnostic imaging were sent for an ultrasound, but the results of the ultrasound were not shared with either the PT or the participant. This allowed us to assess the impact of knowledge of shoulder pathology on response to the intervention.

### Data analysis

Descriptive analyses were performed for all variables. Because there were no published MCID for ROM or strength, we used effect sizes to measure the impact of the intervention. Effect sizes were calculated for ROM and strength measures using the mean difference in scores from baseline to 26 weeks divided by the standard deviation of the mean difference. As per published norms for Cohen’s D test, a small effect size was 0.2–0.49, a moderate effect size was 0.50–0.79, and a large effect size was 0.8 or greater [35]. For the longitudinal analysis, we used a Generalized Linear Mixed Modeling (GLMM) to analyze changes in each of our selected variables over time, using the PROC MIXED procedure in SAS (SAS institute, Version 9.4). The model included Intervals (baseline, 6-, 12- and 26-weeks) as a fixed effect and participant identifier as a random effect to account for repeated measurements within individuals. A compound symmetry covariance structure was specified to model within-participant correlations. Least squares means were computed for each interval, and pairwise comparisons were conducted with confidence limits and p-values adjusted for multiple comparisons. All statistical tests with a p < 0.05 were considered significant. The data tables are provided in Appendix S2.

## Results

### Participants

Of 190 eligible patients, 143 (72%) provided consent with 127 (88.8%) completing at least one follow-up visit and 113 (79%) completing the 26-week research evaluation. Over 85% of participants had rotator cuff pathology confirmed through diagnostic imaging. Knowledge of diagnostic imaging results were associated with referral for a surgical consult, but 50% of those referred for a consult were recommended to return for further PT [16]. Table 1 lists the presenting characteristics of the cohort.

**Table 1:** Presenting characteristics at initial research evaluation.

Variable	All (n = 143)
Demographics	
Mean Age (SD; min, max)	49.0 (10.7; 20, 67)
Females, n (%)	62 (43.4)
Medical History	
Comorbidities	
0 conditions, n (%)	43 (30.1)
1–2 Conditions, n (%)	81 (56.6)
≥ 3 Conditions, n (%)	19 (13.3)
Contralateral Symptoms, n (%)	38 (26.9)
Mean Body Mass Index (SD)	27.4 (4.8)
Medications use, n (%)	87 (60.8)
NSAIDs	11 (7.7)
Opioids	19 (13.3)
Previous Shoulder Injections	
Injury Characteristics	
Dominant Side Affected, n (%)	89 (62.2)
Mean Duration of Symptoms in months (SD)	5.99 (8.32)
Gradual Onset, n (%)	65 (45.5)
Work-related Injury, n (%)	12 (8.6)
Rotator Cuff Pathology Present on Diagnostic Imaging n (%)	108 (85.7)
Employment Status, n (%)	
Full Time/Self-Employed	104 (72.7)
Part-Time	10 (7.0)
Retired	16 (11.2)
Student/Unemployed/Other	13 (9.1)
Work Type, n (%)	
Repetitive above shoulder	33 (23.2)
Repetitive at shoulder	15 (10.6)
Repetitive at waist	14 (9.9)
Desk job	58 (40.8)
No repetitive activity	1 (0.7)
Not applicable (not employed)	21 (14.8)

SD = Standard Deviation; Min = Minimum; Max = Maximum; NSAIDs = Non-Steroidal Anti-Inflammatory Drugs.

### Pain over time

Over time, there was a significant reduction in pain at rest, at night and with activity. Statistically significant and clinically important changes occurred within 12 weeks. Even after completing their PT programs, participants reported ongoing improvements in pain at the final 26-week research evaluation (Table 2).

**Table 2:** Change in pain over time.

Variable	n	Mean VAS Pain Score (95% CI)	Mean Difference from Baseline (95% CI)	P-value
**Pain at Rest**	**127**			**<0.001**
*Baseline*		2.9 (2.5, 3.3)		
*6-weeks*		2.4 (2.0, 2.7)	−**0.5 (−0.9, −0.1)**	
*12-weeks*		1.9 (1.5, 2.3)	**−1.02 (−1.4, −0.6)**	
*26-weeks*		1.2 (0.8, 1.6)	**−1.6 (−2.1, −1.2)**	
**Pain at Night**	**127**			**<0.001**
*Baseline*		4.3 (3.9, 4.8)		
*6-weeks*		3.2 (2.8, 3.7)	**−1.1 (−1.5, −0.6)**	
*12-weeks*		2.4 (2.0, 2.9)	**−1.9 (−2.4, −1.5)**	
*26-weeks*		1.6 (1.1, 2.1)	**−2.7 (−3.2, −2.2)**	
**Pain with Activity**	**127**			**<0.001**
*Baseline*		5.7 (5.3, 6.2)		
*6-weeks*		4.4 (3.9, 4.8)	**−1.3 (−1.8, −0.9)**	
*12-weeks*		3.3 (2.8, 3.7)	**−2.4 (−2.9, −2.0)**	
*26-weeks*		2.5 (2.0, 3.0)	**−3.2 (−3.7, −2.7)**	

VAS = Visual Analogue Scale; CI = Confidence interval.

Generalized Linear Mixed Modeling with Type 3 Tests of Mixed Effects.

Bolded values indicate significance of at least p < 0.05.

### Active range of motion over time

For the most part, active ROM also improved over time, particularly abduction and external rotation in 90 degrees of abduction. Statistically significant changes occurred within six weeks with ongoing improvement reported at 26-weeks, demonstrating moderate effect sizes within 26-weeks for abduction, external rotation and forward flexion. Only internal rotation in 90 degrees abduction showed limited change over time (Table 3).

**Table 3:** Change in active shoulder ROM over time.

Variable	n	Mean Active ROM in Degrees (95% CI)	Mean Difference from Baseline (95% CI)	Effect Size from Baseline to 26-weeks	P-value
**Abduction**	**127**			**0.78**	**<0.001**
*Baseline*		119.7 (113.0, 126.3)			
*6-weeks*		129.9 (123.2, 136.6)	**10.3 (4.7, 15.8)**		
*12-weeks*		137.2 (130.3, 144.1)	**17.6 (11.8, 23.3)**		
*26-weeks*		146.6 (139.5, 153.7)	**27.0 (21.0, 33.0)**		
**Forward Flexion**	**127**			**0.55**	**<0.001**
*Baseline*		143.5 (139.0, 148.0)			
*6-weeks*		149.7 (145.2, 154.3)	**6.2 (2.4, 10.0)**		
*12-weeks*		152.7 (148.1, 157.4)	**9.0 (5.2, 13.1)**		
*26-weeks*		156.6 (151.8, 161.5)	**13.1 (9.0, 17.3)**		
**External Rotation at 0 degrees**	**127**			**0.48**	**<0.001**
*Baseline*		50.1 (46.5, 53.7)			
*6-weeks*		52.3 (48.7, 55.9)	**2.2 (0.4, 4.8)**		
*12-weeks*		55.3 (51.6, 61.5)	**5.2 (2.5, 7.8)**		
*26-weeks*		57.7 (53.9, 61.5)	**7.6 (4.8, 10.4)**		
**External Rotation at 90 degrees**	**127**			**0.51**	**<0.001**
*Baseline*		58.8 (53.9, 63.7)			
*6-weeks*		64.2 (59.2, 69.2)	**5.4 (1.5,9.3)**		
*12-weeks*		68.5 (63.5, 73.5)	**9.7 (5.7, 13.7)**		
*26-weeks*		70.9 (65.8, 76.1)	**12.1 (8.0, 16.3)**		
**Internal Rotation at 90 degrees**	**127**			0.13	**0.03**
*Baseline*		41.0 (37.8, 44.2)			
*6-weeks*		26.9 (9.2, 44.6)	−14.1 (−31.9, 3.7)		
*12-weeks*		39.1 (35.9, 42.3)	−1.9 (−5.0, 1.3)		
*26-weeks*		43.5 (40.1, 46.9)	2.5 (−0.8, 5.9)		

ROM = Range of Motion; CI = Confidence interval.

Generalized Linear Mixed Modeling with Type 3 Tests of Mixed Effects.

Bolded values indicate significance of at least p < 0.05.

### Strength over time

Strength also improved over time, with statistically significant improvements primarily occurring between six- and 12-weeks. Ongoing improvements were reported at 26-weeks with moderate to large effect sizes, particularly for external and internal rotation. Participants regained strength in forward flexion faster than with abduction or shoulder rotation (Table 4).

**Table 4:** Change in shoulder strength over time.

Variable	n	Mean Strength in Newtons (N) (95% CI)	Mean Difference from Baseline (95% CI)	Effect Size	P-value
**Abduction**	**127**			**0.48**	**<0.001**
*Baseline*		21.7 (19.7, 23.7)			
*6-weeks*		21.9 (19.9, 24.0)	0.3 (−1.0, 1.5)		
*12-weeks*		23.9 (21.8, 25.9)	**2.2 (0.9, 3.5)**		
*26-weeks*		25.5 (23.4, 27.6)	**3.8 (2.4, 5.2)**		
**Forward Flexion**	**127**			**0.65**	**<0.001**
*Baseline*		20.8 (19.0, 22.8)			
*6-weeks*		22.5 (20.6, 24.4)	**1.6 (0.3, 3.0)**		
*12-weeks*		24.6 (22.7, 26.5)	**3.7 (2.3, 5.1)**		
*26-weeks*		26.3 (24.3, 28.3)	**5.4 (4.0, 6.9)**		
**External Rotation at 90 degrees**	**127**			**0.65**	**<0.001**
*Baseline*		14.9 (12.9, 17.0)			
*6-weeks*		17.2 (15.1, 19.2)	**2.2 (0.8, 3.6)**		
*12-weeks*		19.3 (17.2, 21.4)	**4..4 (3.0, 5.8)**		
*26-weeks*		21.9 (19.8, 24.1)	**7.0 (5.6, 8.5)**		
**External Rotation at 0 degrees**	**127**			**0.84**	**<0.001**
*Baseline*		18.2 (16.5, 20.0)			
*6-weeks*		19.1 (17.3, 20.8)	0.9 (−0.3, 1.9)		
*12-weeks*		21.3 (19.5, 23.1)	**3.1 (1.9, 4.2)**		
*26-weeks*		22.7 (20.9, 24.5)	**4.4 (3.2, 5.6)**		
**Internal Rotation at 90 degrees**	**127**			**0.71**	**0.03**
*Baseline*		17.7 (15.6, 19.9)			
*6-weeks*		19.9 (17.7, 22.0)	**2.1 (0.7, 3.6)**		
*12-weeks*		22.3 (20.1, 24.5)	**4.6 (3.1, 6.1)**		
*26-weeks*		24.0 (21.8, 26.3)	**6.3 (4.8, 7.9)**		

CI = Confidence interval.

Generalized Linear Mixed Modeling with Type 3 Tests of Mixed Effects.

Bolded values indicate significance of at least p < 0.05.

### WORC score over time

Similar to the other measures, the WORC score also improved over time, with statistically significant changes reported at each measurement point, and clinically important differences occurring within 12-weeks. Again, there was ongoing improvement reported at 26-weeks. (Table 5).

**Table 5:** Change in % in WORC score over time.

Variable	n	Mean % WORC Score (95% CI)	Mean Difference from Baseline (95% CI)	P-value
*Baseline*	**127**	47.5 (43.4, 51.6)		**<0.001**
*6-weeks*		58.4 (54.2, 62.5)	**10.9 (7.4, 14.4)**	
*12-weeks*		68.0 (63.8, 72.3)	**20.5 (16.9, 24.1)**	
*26-weeks*		75.0 (70.6, 79.3)	**27.5 (23.7, 31.2)**	

WORC = Western Ontario Rotator Cuff; CI = Confidence interval.

Generalized Linear Mixed Modeling with Type 3 Tests of Mixed Effects.

Bolded values indicate significance of at least p < 0.05.

### Compliance with program

Overall, 111 (93%) reported that they attended in-person PT, with 110 (92%) performing their home exercises at least four days/week. At the 6-week evaluation, 101 (90%) participants performed proper exercise technique for the research evaluator.

## Discussion

This study aimed to determine the impact of a criteria based, progressive exercise PT program that included both supervised PT and home exercises for patients with RCRSP over 12-weeks with an evaluation period of 26-weeks. Overall, participants reported statistically significant and clinically relevant reductions in pain (p < 0.001) and HRQL (p < 0.001) and demonstrated significant improvements in shoulder ROM (p < 0.001) and strength (p < 0.001) with moderate to large effect sizes within 26-weeks. Self-reported compliance and demonstration of correct exercise technique were over 90%.

Reductions in pain over time, our primary outcome, occurred in all circumstances (rest, at night and with activity) at every evaluation period (6-weeks, 12-weeks and 26-weeks). The most significant changes were reported between baseline and six weeks, aligning with the program’s primary goal for Phase I: reducing/managing pain. Pain non-response at six weeks may signal a need for further investigation and/or incorporating additional pain management strategies to the exercise program, as deemed appropriate by the treating PT [19,22,36–38]. Significant reductions in pain continued after six weeks, including after program cessation, where further pain reduction was reported at 26-weeks.

Secondary outcome measures of active shoulder ROM and strength also improved. In particular, abduction and external rotation motion at 90 degrees of abduction significantly improved between baseline and six weeks with continued improvements demonstrating moderate effect sizes of the intervention up to 26-weeks. In addition to decreasing pain, the first six weeks of the exercise program aimed to increase shoulder girdle ROM and initiate RC and scapulothoracic strength exercises. As pain decreased during Phase I, patients could perform mobility and strength exercises with greater ease and effectiveness. Individualizing dosage, intensity of exercise and educating patients regarding pain expectations may have allowed participants to complete their exercises while managing their pain. Curiously, internal rotation at 90 degrees of abduction was the only active ROM that did not change over time. Further study is required but this is often a position of symptomatic impingement for patients with RCRSP. Therefore, pain may have influenced participants’ willingness to go into full internal rotation. In addition, measuring active internal rotation reliably in this overhead position is difficult because the scapula is not stabilized. Following ROM improvements, strength improved most significantly between six and 12 weeks with further improvement and moderate to large effect sizes seen at 26 weeks. This aligns with Phase II’s primary goals to increase RC and scapulothoracic muscle strength. Once patients can actively move their shoulder with less pain, the focus of PT shifted to strengthening the RC and scapulothoracic muscles. Participants regained strength in forward flexion faster than abduction or external rotation, which is not surprising, given that abduction and external rotation are generally more affected with RCRSP, with increased pain and weakness, leading to a slower rate of progress. The improvements in mobility and strength, in combination with reductions in pain, also led to significant improvements in participants’ self-reported improvements in the WORC score over time.

Progressive exercises that address rotator cuff, scapular and adjacent spine strength and mobility are considered first-line treatments in managing RCRSP as they appear to be similar to surgery in clinical effectiveness and superior in cost-effectiveness [6,13,19–22]. Pieters et al, in their 2020 update of systematic reviews examining the effectiveness of PT interventions in patients with RCRSP [20], yielded 7/16 (44%) relevant studies with high- or moderate-level evidence supporting exercise therapy as part or all of the PT treatment plan. Many of the included RCTs and systematic reviews did not provide details regarding exercise types, frequency, dosage, criteria for progression, program duration or determinants for surgical referral. This is a common limitation in shoulder rehabilitation literature, making it difficult to interpret results and replicate programs in clinical practice [6,19–22]. A major strength of our study is that we addressed many of these limitations by utilizing a standardized PT program that provided detailed information, including progression criteria and a proven algorithm for referring non-responsive patients for an orthopedic surgical consult [16], but did not create a ‘one size fits all’ program.

PTs delivering treatment received in-person educational sessions and had access to on-line resources, including videos. Individual patient dosage determinations, including education on PRE and pain expectations, correct technique, home exercise compliance and progression over 12 weeks were emphasized. The exercise program was based on a standardized non-operative shoulder guideline that addressed key shoulder principles, along with goals in each phase and criteria for progression to the next phase, which were not solely time-based, recognizing the heterogeneity of RCRSP. This approach standardized treatment, while still allowing customization for participants, including progression based on response to treatment.

Using the research assessor to determine patient compliance, in addition to self-report, was another strength of our study. Patients were given home exercise parameters and advised to perform exercises four times/week. At each assessment period, participants were required to demonstrate their exercises to the research assessor to demonstrate their understanding and ability to perform their home exercises independently. Patient compliance was very good, with >90% demonstrating correct exercise techniques at the six-week evaluation. Follow-up of our original cohort was 79% at the final 26-week evaluation period.

Dosage recommendations emphasized working to fatigue but stopping if/when correct exercise form failed and/or sharp pain was experienced. The high level of compliance attained may be related to the consistent reinforcement regarding the importance of home exercise in combination with the education on PRE expectations and anticipated pain responses; this approach may have helped patients perform home exercises confidently and consistently. While pain is a predominant symptom associated with RCRSP, and an important component of treatment, the entirety of the specific patient’s kinetic chain, shoulder girdle mobility, strength and stability need to be addressed when designing and implementing appropriate programs [6,20,22,37]. Thus, shoulder rehabilitation programs need to be long enough to address the multifactorial impairments associated with RCRSP [36,39]. Participants typically attended in person PT every one to two weeks initially with a gradual increase in time between sessions as patients became more confident with their home program and demonstrated improved ROM, strength, function and decreased pain. In person PT sessions continued until 12 weeks, and were largely used to reassess, check exercise technique and progress exercise difficulty as able. PTs were encouraged to address pain management as needed as an adjunct to the exercise program. However, as a non-comparative study, further evaluation is needed, using a randomized design to determine the optimal duration and delivery method of a progressive shoulder exercise program.

### Limitations

The main study limitation is that we used an observational study design, so we are unable to determine if the length of our exercise program (12 weeks) and combined in-person and home exercise delivery model achieved better results than other approaches. Further study to investigate and compare the program to usual care and against different program lengths and delivery models is warranted. We also did not confirm rotator cuff pathology until completion of the study for those participants who did not have diagnostic imaging performed until after entering the study, but 85% of our participants were eventually confirmed to have RC pathology. Our previous evaluation found that knowledge of shoulder pathology prior to treatment did not affect reported outcomes, but did increase referral to a surgeon; however, 50% of those referred to the surgeon were recommended to continue the PT program [16]. Additionally, 11% of the original cohort was not included in the final 26-week follow-up period because they required surgery, which may have led to an overestimation of the program impact. Participant compliance could have also been further strengthened through use of specific exercise logs. Further, allowing for individual program customization limited our ability to report the number and duration of each visit; however, results suggest that clinicians were able to use the shoulder guidelines to deliver a standardized treatment with individualization. Finally, this was a planned secondary descriptive analysis, so we did not perform a power calculation for this secondary objective. Instead, we focused on reporting our findings relative to published MCIDs, or effect sizes where MCIDs were not available.

## Conclusion

Our criteria-based progressive exercise PT program that incorporated in-person and home exercise components was effective in significantly reducing pain, improving active ROM, strength and HRQL. Program details were well communicated to treating PTs and participants with emphasis placed on working towards similar goals while allowing customization based on individual patient presentation. Attention to dosage determination, exercise intensity, proper technique as well as patient education regarding pain expectations were believed to contribute to the > 90% compliance result.

## Supporting information

S1 Appendix This is the referral algorithm. (DOCX)

S2 Appendix This file holds the data tables. Table 1 does not contain study IDs and sex and gender have been listed as separate tables as the cohort is too small to ensure that a study participant cannot be identified (i.e., cells of <5 can be produced). However, Table 1 can be reproduced with the data provided and is independent of information reported in Tables 2–5.
(XLSX)
