# Supplementary material for: A standardized criteria-based progressive shoulder exercise program is effective in managing rotator cuff-related shoulder pain: A prospective cohort study
Source: PLoS One. 2025 Jul 23;20(7):e0328728. doi: 10.1371/journal.pone.0328728 (PMC12286389; doi:10.1371/journal.pone.0328728)
Supplement: S1 Appendix — (DOCX) [file pone.0328728.s001.docx]

**Supplemental Appendix 1: Referral Algorithm for Intervention Physical Therapists**
